# Accurate Sparse-Projection Image Reconstruction via Nonlocal TV Regularization

**DOI:** 10.1155/2014/458496

**Published:** 2014-01-27

**Authors:** Yi Zhang, Weihua Zhang, Jiliu Zhou

**Affiliations:** College of Computer Science, Sichuan University, No. 24, South Section 1, Yihuan Road, Chengdu 610065, China

## Abstract

Sparse-projection image reconstruction is a useful approach to lower the radiation dose; however, the incompleteness of projection data will cause degeneration of imaging quality. As a typical compressive sensing method, total variation has obtained great attention on this problem. Suffering from the theoretical imperfection, total variation will produce blocky effect on smooth regions and blur edges. To overcome this problem, in this paper, we introduce the nonlocal total variation into sparse-projection image reconstruction and formulate the minimization problem with new nonlocal total variation norm. The qualitative and quantitative analyses of numerical as well as clinical results demonstrate the validity of the proposed method. Comparing to other existing methods, our method more efficiently suppresses artifacts caused by low-rank reconstruction and reserves structure information better.

## 1. Introduction

Computed tomography (CT) has still been a widely used medical imaging technology for clinical diagnosis. However, according to the recent reports, the risk of overhigh radiation has caused social attention. It is well known that it is harmful for human body to expose to heavy radioactive source. As a result, the problem which arises is, when CT scans are inevitable, and how can we reduce the radiation dose without losing the imaging quality?

To deal with this issue, many technologies which can be categorized into two groups were proposed. The first one is to lower the configuration parameters of X-ray. The key step is to reduce the milliampere seconds (mAs) or kVp parameter; however, the quantum noises also appear. Many methods were proposed to suppress the quantum noises [1–6]. The vital problem of this kind is that under the situation of low operational current or voltage, when high density objects, such as metal implants or bones exit, the severe attenuation of X-rays allows only a limited number of photons to reach detectors. As a result, new artifacts will be introduced in the reconstructed image. The artifacts spread through the whole image, which contaminate the imaging quality. Therefore, the second category does not change the energy of X-ray. Instead of that, reducing the projection number which is also called sparse-projection reconstruction is another way to achieve this goal. However, due to the lack of projection views, streak artifacts will severely affect the imaging quality. This topic can be viewed as an ill-posed inverse problem which has provoked many studies about it. In this paper, we will focus on sparse-projection reconstruction.

Compressive sensing (CS) is an efficient method to handle sparse-projection reconstruction [7, 8]. The main idea of CS is very similar to sparse-projection reconstruction and both of them manage to recover the complete signals from a severe undersampling. Many studies have been done following such concepts. In [9], Chen et al. considered a prior image as a prior knowledge (PICCS). Based on the fact that in many CT imaging applications some physical and anatomical structures and the corresponding attenuation information of the scanned object can be a priori known, Rashed and Kudo presented a statistical iterative reconstruction (SIR) by incorporating this prior information into the image reconstruction objective function [10]. Ma et al. introduced nonlocal means into low-dose reconstruction with a precontrast scan [11]. The most famous model with CS theory is total variation (TV) based method called ASD-POCS which is firstly proposed by Sidky et al. [12, 13]. They introduced TV into algebraic reconstruction technique (ART) to suppress the artifacts caused by the limitation of projection views. Following this idea, the same group replaced *L*_1_ norm with *L*_*p*_ norm in the minimization function [14]. Ritschl et al. proposed a step-size-adaptive method based on TV to eliminate the dependence on the raw data consistency [15]. To improve the convergence and efficiency of TV based minimization methods, Yu and Wang constructed a pseudoinverse of discrete gradient transform (DGT) and adapted a soft-threshold filtering algorithm [16]. Lu et al. proposed a novel algorithm for image reconstruction from few-view data. It utilizes the simultaneous algebraic reconstruction technique (SART) coupled with dictionary learning, sparse representation, and TV minimization on two interconnected levels [17]. Although TV based methods have achieved efficient results, TV suffered from the notorious blocky effect which obstructs the clinical practice of TV. To overcome this disadvantage; many efforts were made [18–20]. Zhang et al. combined classical TV with a high-order norm to suppress the blocky effect [21]. Fractional calculus was introduced into ASD-POCS model [22]. By adjusting the order of fractional-order TV norm, blocky effect can be reduced to an acceptable level without increasing much computational cost. The reason of this side effect should put the blame on the basic assumption of TV that the images are piecewise constant and TV is a local-related computation.

In this paper, we propose a nonlocal TV based model to deal with the sparse-projection image reconstruction. First, we will review the classical TV based model in the next section. The details of our method are represented in Section 3. Numerical and clinical experiment results are demonstrated in Section 4. The discussion and conclusion are given at the end of this paper.

## 2. A Brief Review about TV Based Image Reconstruction Method

Because our method is an extended version of TV based image reconstruction, in this section, we first give a brief description of this method. Given a 2-dimensional image *u* = *u*(**x**) = *u*_*x*_1_,*x*_2__, whose size is *M* × *N*, *x*_1_ ∈ [1, *M*] and *x*_2_ ∈ [1, *N*], for any *u*, the gradient operator is defined as

(1) $$\begin{array}{c} \nabla u=\left(\Delta_{x_{1}}u,\Delta_{x_{2}}u\right), \end{array}$$

where Δ_*x*_1__ and Δ_*x*_2__ are the first-order differential operators along *x*-axis and *y*-axis, respectively. Δ_*x*_1__ and Δ_*x*_2__ can be represented as

(2) $$\begin{array}{c} \Delta_{x_{1}}u=u_{x_{1},x_{2}}-u_{x_{1}-1,x_{2}},\\ \Delta_{x_{2}}u=u_{x_{1},x_{2}}-u_{x_{1},x_{2}-1}. \end{array}$$

**Figure 1:**
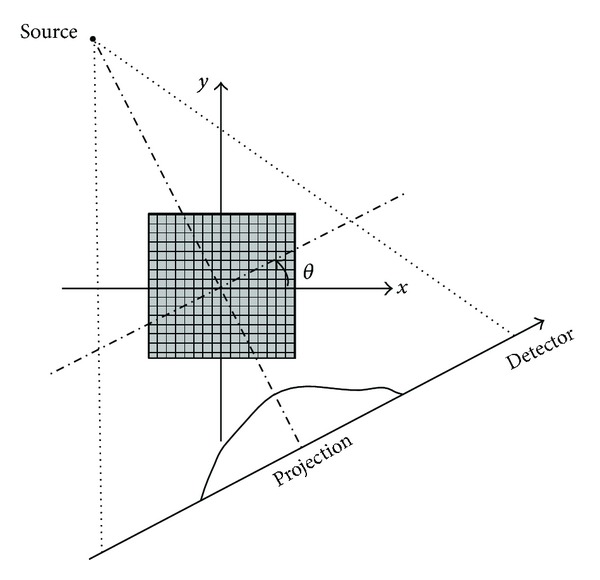
Fan-beam CT geometry configuration.

In traditional CT imaging problem, the sampling procedure can be considered as a discrete linear transform

(3) $$\begin{array}{c} Au=f, \end{array}$$

where *A* is the system matrix which is comprised of *I* row vectors and *f* = (*f*_1_, *f*_2_,…, *f*_*I*_)^*T*^ is the measurement vector. The individual elements of the system matrix are *A*_*ij*_, where *i* = 1,2,…, *I* and *j* = 1,2,…, *J*. It is obvious that *M* × *N* = *J*. Without losing generality, the fan-beam projection geometry can be demonstrated in Figure 1.

To solve the linear system in (3), the TV based image reconstruction algorithm which was used to deal with the sparse-projection limitation is to optimize the following problem [12, 13]:

(4) $$\begin{array}{c} \min{||u||}_{\text{TV}}\;\text{subject}\;\text{to}\;u\geq 0,\;Au=f, \end{array}$$

where ||*u*||_TV_ can be considered as a *L*_1_ norm of the first-order gradient image ∇*u*. The TV based algorithm combined the steepest decent method and the projection on convex sets (POCS) to achieve the solution of (4) iteratively.

## 3. The Proposed Nonlocal TV Reconstruction Method

The locality of TV is the main factor which causes blocky effect and it is the motivation we introduce nonlocal TV based method for alleviating this phenomenon. The minimization problem of CT image reconstruction can be formulated as

(5) $$\begin{array}{c} \min J\left(u\right)\;\text{subject}\;\text{to}\;u\geq 0,\;Au=f, \end{array}$$

where *J*(*u*) is the regularization term and other symbols are with same meanings as (4). The key part is how to choose *J*(*u*). In TV based model, *J*(*u*) = *J*_TV_(*u*) = ||*u*||_TV_ and in our proposed method, *J*(*u*) = *J*_NLTV_(*u*) = ||*u*||_NLTV_. Inspired by [23–26], we define ||*u*||_NLTV_ as

(6) $$\begin{array}{c} {||u||}_{\text{NLTV}}=\int_{\Omega }\left|\nabla_{\text{NLTV}}u\left(x\right)\right|dx, \end{array}$$

where the nonlocal gradient ∇_NLTV_ *u*(**x**) is defined as the vector of all partial differences ∇_NLTV_ *u*(**x**, ·) at **x** such that

(7) $$\begin{array}{c} \nabla_{\text{NLTV}}u\left(x,y\right)=\left(u\left(y\right)-u\left(x\right)\right)\sqrt{w\left(x,y\right)},\;\forall y\in \Omega . \end{array}$$

So we obtain the minimization function

(8) min⁡ ∫Ω∫Ω(u(x)−u(y))2w(x,y)dydx,subject  to u≥0, Au=f,

where *w*(**x**, **y**) is a weighting function to compute the similarity between vectors **x** and **y**. *w*(**x**, **y**) is defined as [25]

(9) $$\begin{array}{c} w\left(x,y\right)=\exp\left(-\frac{G_{\alpha }*{\left|u(n(x))-u(n(y))\right|}^{2}}{h^{2}}\right), \end{array}$$

where *G*_*α*_ is the Gaussian kernel with standard deviation *α* and *h* is filtering parameter controlling the decay of the exponential function. Generally, *h* is determined by the noise level. *n*(**x**) and *n*(**y**) denote the two local similarity neighborhoods (named patch windows) centered at the pixels **x** and **y**, respectively, and we only compute the weights in a semilocal searching window for each pixel. To simplify the optimization problem, we reformulate (5) as

(10) $$\begin{array}{c} E\left(u\right)=\int_{\Omega }\left|\nabla_{\text{NLTV}}u\left(x\right)\right|dx+\frac{\lambda }{2}{||Au-f||}_{2}^{2}, \end{array}$$

where *λ* is a parameter to control the balance between regularization and fidelity terms.

Then, we use the gradient descent to update the solution by the Euler-Lagrange equation of (10):

(11) $$\begin{array}{c} u_{t}=-Ru+\lambda A^{*}\left(f-Au\right), \end{array}$$

where A* is the adjoint of *A* and

(12) Ru =−∫Ω(u(y)−u(x))w(x,y)     ×[1|∇NLTVu(x)|+1|∇NLTVu(y)|]dy.

Our proposed method for sparse-projection image reconstruction can be summarized by the following steps:

(a) initialization of performance parameters and stopping criteria;
(b) outer loop for *p* = 1,2,…, *P*: reconstruction with SART algorithm:
  (13) $$\begin{array}{c} u_{j}^{p+1}=u_{j}^{p}+\frac{1}{A_{+,j}}\overset{I}{\underset{i=1}{\sum }}\frac{A_{i,j}}{A_{i,+}}\left(f_{i}^{0}-f_{i}^{p}\right),\\ A_{i,+}=\overset{J}{\underset{j=1}{\sum }}A_{i,j},\;\text{for}\;i=1,\ldots ,I,\\ A_{+,j}=\overset{I}{\underset{i=1}{\sum }}A_{i,j},\;\text{for}\;j=1,\ldots ,J; \end{array}$$
(c) inner loop for *q* = 1,2,…, *Q*: NLTV gradient descent method:
  (14) uq+1=−Ruq  +λA∗(f−fp);
(d) repeat beginning with step (b) until the stopping criteria are satisfied.

## 4. Numerical and Clinical Results

In this section, to validate and evaluate the proposed method, numerical and clinical experiments are performed. In the numerical experiments, Shepp-Logan phantom was used and we simulated X-ray projections using Siddon's ray-driven algorithm [27] in fan-beam geometry. The source to rotation center distance is 40 cm and the detector to rotation center is 40 cm. The image array is 20 × 20 cm^2^. The detector whose length is 41.3 cm is modeled as a straight-line array of 512 detector bins. All the tests are performed by MATLAB on a PC with Intel i7-3770 CPU 3.40 GHz and 8 Gb RAM. In clinical experiment, we applied our method to a typical CT slice of a human chest. All the scans were performed on a Siemens SOMATOM Sensation 64 MSCT scanner (Siemens Medical System, Erlangen, Germany) except for the Shepp-Logan (S-L) phantom. The voltage and current were 120 kVp and 200 mA with a slice thickness of 1 mm. To get a good visual effect of our method, we compare the proposed method to FBP, EM, and ASD-POCS. The reconstruction results are also quantitatively evaluated in terms of RMSE and MSSIM whose computational definitions are given in [28, 29]. In all the experiment results, the main parameters were set as *α* = 1, the search window size *N*_*s*_ = 5 × 5, the patch window size *N*_*p*_ = 21 × 21, *λ* = 0.1, *P* = 100, and *Q* = 20. Moreover, we chose *h* to be the estimated noise variance in the filtered back projection image. We used a wavelet based noise estimation model introduced by Donoho and Johnstone in these experiments [30].

### 4.1. Phantom Cases

In this section, the numerical experiment results are given. The results were performed under ideal condition, in which projection data were generated numerically without adding noise. To demonstrate the performance of our method, Shepp-Logan phantom was uniformly sampled with 30 over 360 degrees. The iteration numbers of EM, ASD-POCS, and NLTV were simply set to 100.

**Figure 2:**
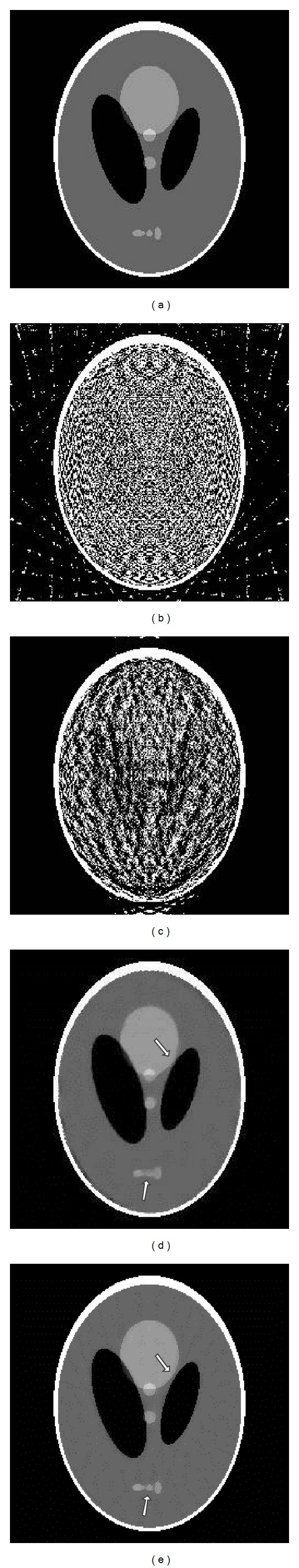
Reconstruction results of Shepp-Logan phantom by different methods. (a) Original, (b) FBP, (c) EM, (d) ASD-POCS, and (e) NLTV.

The reconstruction results of Shepp-Logan phantom are given in Figure 2. In Figure 2(b), it is obvious that, due to incompleteness of projection data, classical FBP cannot achieve an acceptable solution. Although EM is a widely used method, its result in Figure 2(c) is not satisfactory. The whole image is fulfilled with severe artifacts and it is difficult to recognize any parts of the phantom. In Figures 2(d) and 2(e), ASD-POCS and NLTV suppress most of the artifacts. ASD-POCS recovers all the virtual organs except some structural parts. As we marked with white arrows in Figures 2(d) and 2(e), when ASD-POCS dealt with regions full of small structure information, oversmoothing effect appeared. Different parts with very small intervals are obscure and some edges are also blurred. Compared to ASD-POCS, NLTV can reduce the impact to a certain extent. The edges in Figure 2(e) are well kept and three oval organs in the bottom of image are more distinguishable. Meanwhile, the corresponding quantitative evaluations are shown in Table 1. Statistically, NLTV yields better RMSE and MSSIM than those of other methods, but the computational cost is much higher.

### 4.2. Clinical Cases

**Figure 3:**
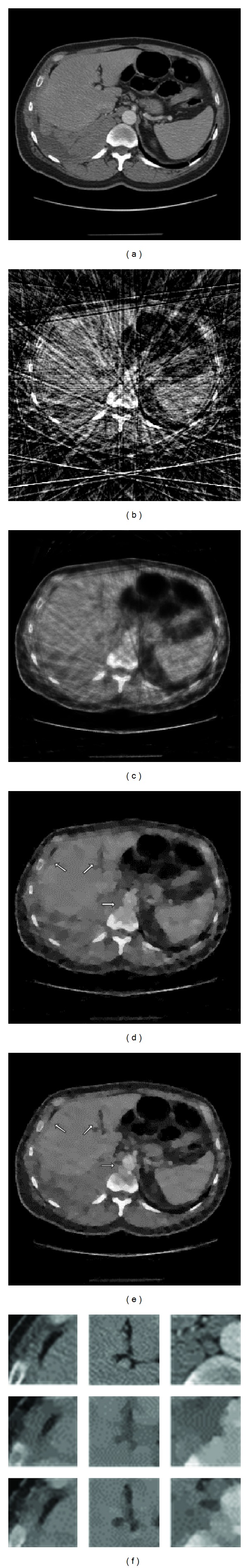
Reconstruction results of chest image by different methods. (a) Original, (b) FBP, (c) EM, (d) ASD-POCS, (e) NLTV, and (f) zoomed parts of original image, ASD-POCS, and NLTV. First row is original images, the second row is ASD-POCS results, and the third row is NLTV results.

In this section, we validated the proposed method on a clinical case which was scanned by a Siemens SOMATOM Sensation 64 MSCT scanner (Siemens Medical System, Erlangen, Germany). The voltage and current were 120 kVp and 200 mA with a slice thickness of 1 mm. To demonstrate the effectiveness of our method, the results processed by FBP, EM, and ASD-POCS are given for comparison. The full scanned image with 550 views is used as a reference image. The image is downsampled uniformly to 20 views, about two-tenths of the original dataset. The iteration numbers of EM, ASD-POCS, and NLTV were set to 100 uniformly. The reconstructed images are displayed in Figure 3.

It is obvious that FBP and EM cause considerable streak-like artifacts in Figures 3(b) and 3(c) and the structure information of tissues is of terrible vision. ASD-POCS and NLTV dispel most of the artifacts, so that most of the tissues can be seen in Figures 3(d) and 3(e). However, in Figure 3(d), the boundaries between different tissues are blurred and the blocky effects are visible in the tissues. Three examples are given in Figures 3(d) and 3(e) by white arrows. NLTV kept most of structure information well and much less side effect. The zoomed parts indicated by white arrows are displayed in Figure 3(f). The quantitative evaluations are shown in Table 2. It can be seen that the results show the coherence with the results of numerical phantom. NLTV obtains better RMSE and MSSIM than other methods but suffers from larger computation overheads.

## 5. Discussion and Conclusion

With the development of modern medical imaging technologies, CT has been playing an increasing important role in clinical analysis. Image reconstruction with sparse projection is one of the most efficient ways to lower the dose the patients will endure. CS is a powerful tool to deal with this problem. CS has proved that a complete signal can be recovered, while a sparsifying transform exists. In this situation, Nyquist sampling theory may not be fit. TV is widely used as an efficient sparsifying transform and it can be introduced into many topics in CT reconstruction, such as sparse projection, limited-angle, and interior reconstruction. Although TV is popular, the blocky effect in homogeneous regions limits the applications in clinical practice. The main reason for this phenomenon is that TV is neighborhood based and there is no global information involved. This will lead to loss of structure and texture information. To solve this problem, we introduce NLTV into medical imaging and give its application in sparse-projection reconstruction. NLTV calculates the weights by searching in all the image patches and it avoids being dependent only on neighboring pixels. The experimental results show that the presented NLTV method can yield more significant performance gains than the existing methods, including FBP, EM, and ASD-POCS, in terms of visual effect and different measurement metrics.

There are several parameters in our methods. All of them should be determined manually, namely, the search window size *N*_*s*_, the patch window size *N*_*p*_, the filtering parameters *α*, and the regularization scale parameter *λ*. Note that, all the parameters are application related. In our purpose, a bigger *N*_*s*_ theoretically means that more similarity information will be acquired. By extensive experiments, *N*_*s*_ = 21 × 21 and *N*_*p*_ = 5 × 5 will be appropriate settings for effective noise and artifact suppression while maintaining computational efficiency. For the other parameters, *α* and *λ*, in our simulations, we simply select the best average configuration based on the results obtained with a broad range of parameter values manually in terms of visual inspection and quantitative measurements. Due to the computational cost of the proposed method, an adaptive mechanism will be useful and in the future work, we will focus on this problem.

Another concern is the computation cost of the proposed method. As a result of introducing global patch distance computation, the computational burden is much bigger than other iteration based methods. However, with the rapid development of storage hardware, the processing time will not be main obstacle and also the proposed method can be implemented on PC clusters or on graphic processing units (GPU), which will make it feasible for practical application.

In conclusion, in this paper, we present a novel sparse-projection image reconstruction method using nonlocal total variation. After experiments on numerical phantoms and clinical cases, the proposed method shows better performance than several commonly used methods with respect to both quantitativeness and qualitativeness. Although the computational cost of this method is larger than current methods, there are several methods that can accelerate the processing speed. It will be convenient to implement and add to modern CT systems. The optimization of adaptive parameter selection and acceleration is another concern in our future work.

## Figures and Tables

**Table 1:** Evaluations of numerical phantom reconstruction.

	RMSE	MSSIM	Iterations	Time (s)
FBP	0.4073	0.4568	—	**0.0290**
EM	0.1548	0.7896	100	2.3878
ASD-POCS	0.0062	0.9932	100	5.9048
NLTV	**0.0022**	**0.9976**	100	69.87

**Table 2:** Evaluations of clinical image reconstruction.

	RMSE	MSSIM	Iterations	Time (s)
FBP	0.2260	0.6779	—	**0.0310**
EM	0.0581	0.7190	100	2.9973
ASD-POCS	0.0562	0.7371	100	6.1286
NLTV	**0.0403**	**0.8214**	100	84.53
